# ‘Who Am I?’: An Exploratory Study of the Relationships Between Identity, Acculturation and Mental Health in Autistic Adolescents

**DOI:** 10.1007/s10803-019-04016-x

**Published:** 2019-04-19

**Authors:** Lily Cresswell, Eilidh Cage

**Affiliations:** Department of Psychology, Royal Holloway, University of London, Egham, Surrey TW20 0EX UK

**Keywords:** Adolescence, Identity, Acculturation, Mental health

## Abstract

Autistic adolescents are at increased risk of mental health difficulties. One potential factor contributing to this is identity development, although this hypothesis has been little explored. These adolescents also have to consider how autism forms their identity, a process called acculturation. This exploratory study examined the relationships between identity, acculturation and mental health in autistic adolescents. Twenty-four participants completed measures investigating identity, acculturation and mental health. Findings suggested mental health was not related to personal identity. Mental health scores did not differ between acculturation groups, however those aligned to non-autistic culture tended to generate more positive self-statements than those aligned to neither culture. These findings suggest autistic adolescents should be encouraged to explore autistic culture and supported in constructing their identity.

Autism is a neurodevelopmental condition, characterised by difficulties with social communication and interaction, as well as repetitive patterns of behaviour, focused activities or interests and sensory sensitivities (American Psychiatric Association 2013). Adolescence may be especially difficult for autistic^1^ individuals, as social expectations increase and relationships become more complex (Adreon & Stella 2001; Rosenthal et al. 2013). McGovern and Sigman (2005) noted that the desire for social relations increases for many autistic individuals during adolescence, but this desire often goes unmet due to social difficulties (Carter et al. 2014). Further, autistic adolescents are common targets of bullying at school (e.g. Cappadocia et al. 2012), with this group being up to four times more likely to experience bullying than their non-autistic peers (Sterzing et al. 2012). Autistic adolescents may therefore find it challenging to make sense of where they fit into society when peer rejection is common.

Research has shown that autistic people are at higher risk of developing mental health conditions, particularly anxiety and depression (e.g. Tantam 2000; Ghaziuddin et al. 2002), and this increased vulnerability is particularly prominent during adolescence (Vickerstaff et al. 2007; Lecavalier 2006). Given this prevalence amongst autistic adolescents, it is important to examine factors that might contribute to the development of these difficulties. One key task that takes place during adolescence is identity development, whereby an individual begins to question and distance themselves from the expectations, values and identities handed down to them from their parents (Marcia 1980).

Identity can be defined as the way a person understands and views him or herself, and is often viewed by others (Holland 2001). Identity status theory (Marcia 1980; Kroger et al. 2010a, b) purports that one’s sense of identity is determined by the choices and commitments made regarding certain personal and social traits. Marcia (1980) suggested that a well-developed identity comprises of a sense of one’s strengths, weaknesses, and individual uniqueness. Marcia (1980) proposed two processes of identity development: exploration and commitment. Exploration refers to a period of thinking through and testing various roles, while commitment refers to the degree of personal investment the individual expresses in a course of action or belief (Kroger and Marcia 2011).

Erikson (1968) introduced the idea that a relationship exists between identity development and mental health, which is still considered useful in understanding mental health today (Côté 2018). Research using non-autistic samples shows that adolescents with high anxiety struggle more with identity development (Crocetti et al. 2008) and those who have not made a commitment tend to have higher depressive symptoms (Meeus et al. 2012). A growing body of empirical studies that use the identity status model indicate that developing a synthesized sense of identity facilitates wellbeing (Waterman et al. 2010) and protects against internalizing symptoms (Ritchie et al. 2013) and externalizing problems (Crocetti et al. 2013).

The process of identity development could be particularly difficult to navigate for autistic individuals (Gill 1997; Ratner & Berman 2015). There is evidence that autistic children and adolescents tend to have differences in their self-perceptions, with young autistic people being less likely to perceive themselves as socially or physically competent and reporting poorer ratings of global self-worth compared to non-autistic peers (Capps et al. 1995). Awareness of being different, and of being potentially stigmatised by others, could have an impact on the development of the self-concept and self-esteem of the individual, a finding which has been demonstrated amongst deaf adolescents (Cornell & Lyness 2005) and highly gifted students (Wang & Neihart 2015). The individual may end up sharing the negative representations that others associate with their ‘differentness’ (Corrigan et al. 2005; Link et al. 2014). If identity development is more difficult for autistic individuals, then it is important to examine how identity development may relate to mental health, given the previously identified links between difficulties with identity development and mental health (e.g., Crocetti et al. 2008; Meeus et al. 1999).

Autistic adolescents not only have to negotiate forming their personal identity, but they also start to question their social identity and where they fit in, in terms of their minority group status within the majority culture of non-autistic peers (Ozonoff et al. 2002), asking questions such as “are they just going to reject me?” of non-autistic peers (Acker et al. 2018). Social Identity Theory (Tajfel & Turner 1979; Islam 2014) assumes that one part of the self-concept is defined by belonging to certain social groups. If group membership provides individuals with a sense of meaning, purpose, and belonging, it can have positive psychological consequences (Haslam et al. 2009). Shared social identity can be protective against the negative consequences of marginalisation, by members of the disadvantaged group coming together to buffer the effects of discrimination (Blaine and Crocker 1995; Carrasco et al. 2017; Postmes and Branscombe 2002).

It is important to consider how autistic people incorporate the fact they are autistic into their social identity. Culture can be defined as a system of meanings through which people organise and make sense of their lives. There has been recent development of the idea that autistic people are building a culture around the ways of speaking, thinking, and acting that come naturally to autistic people (Davidson 2008a, b; Dekker 1999). These different modalities of expression provide important clues to systems of meaning on which inferences regarding autistic culture can be made. Autistic culture parallels the emergence of deaf culture (Halpern 1996), with both being supportive communities focused on the distinctive issues and experiences related to being autistic or deaf (Gobbo & Shmulsky 2016).

Acculturation refers to the process of cultural and psychological change that occurs following the meeting between cultures (Sam & Berry 2010). Although acculturation typically refers to the process of identity change after moving to a new country, we posit that an analogous process occurs when navigating self-identity as an autistic person in a non-autistic world. Acculturation in the context of autistic culture could therefore be measured using Berry’s (1986, 2005) model, applied to the experiences of autistic people and their ways of speaking, thinking and acting. Berry (1986, 2005) describes four acculturation types: marginalised (alignment to neither cultural group), bicultural (alignment to both cultural groups), assimilated (alignment to majority group, rejecting own minority group) and separated (alignment solely to those from own minority group). Whether to align oneself to autistic culture or the majority (non-autistic) group, might be a complex negotiation faced by many autistic adolescents. It could be considered a process of identity exploration, leading towards commitment when the individual aligns themselves to autistic culture (or not), as per Marcia’s (1980) identity status theory.

There is little evidence on how autistic people navigate their identity in terms of acculturation to autistic culture. Kenny et al. (2016) found that most autistic adults and their families preferred identity-first terminology (‘autistic’) to person-first terminology (‘person with autism’), as they stated that autism forms an integral part of the person. The neurodiversity movement advocates self-identification as autistic and viewing autism as a positive identity that needs no cure (Chamak et al. 2008; Ortega 2009). Qualitative evidence suggests that some autistic adolescents take pride in being autistic (Cage et al. 2016), and some view autism as part of ‘who they were’, and they had come to accept their differences (Humphrey & Lewis 2008).

However, there is also evidence to suggest that some autistic young people distance themselves from autistic culture (Baines 2012; Huws and Jones 2010) and hold negative perceptions of their differences, along with showing concern for not ‘fitting in’ (Humphrey & Lewis 2008). Carrington and Graham (2001) interviewed two autistic adolescents about their experiences and found that they were more aware as they grew older of their ‘differentness’ and they wanted to fit in but did not know how. The emotional stress associated with the need to belong (Baumeister & Leary 1995; Fiske 2009) suggests it may be important for the wellbeing of autistic adolescents to have a positive sense of group belonging. In autistic adults, Cooper et al. (2017) conducted a large-scale study that measured self-esteem, depression, anxiety and autism identification. Results indicated that less autism identification related to more anxiety and depression symptoms, which was mediated through increases in collective self-esteem (perceived positivity of autism identity) and personal self-esteem. This finding suggests having a positive autistic social identity might offer a protective mechanism against psychological difficulties for autistic adults.

Given that identity development has been found to relate to mental health outcomes in the general population (Crocetti et al. 2008; Meeus et al. 2012), and that identity concerns are prevalent amongst autistic adolescents (Baines 2012; Cage et al. 2016; Huws and Jones 2010), it is worth further exploring the relationship between identity development and mental health in autistic adolescents. The current study therefore investigates acculturation, exploring not only how autistic adolescents identify with and align themselves to autistic culture, but also how they relate to non-autistic culture. First, we propose that there will be a relationship between personal identity and mental health in autistic adolescents. Second, we predict a relationship between acculturation type and mental health. Finally, it is anticipated that there will be relationship between acculturation type and positive or negative self-concept. It is predicted that commitment to one cultural group (marginalised or separated) will relate to better outcomes of mental health and positive self-concept.

## Methods

### Participants

Participants were recruited through mainstream secondary schools in London, UK, and through a community service for autistic adolescents and their parents, via direct contact with senior members of staff, who disseminated the project information and consent forms to parents. After parental consent was obtained, the researcher arranged to meet each consented young person, either at home or at school, to explain the project and obtain their informed written consent. To meet eligibility, individuals had to be aged 12–18 and have a diagnosis of an Autism Spectrum Condition (ASC). Individuals with a diagnosed learning disability were not eligible for participation due to verbal complexity of the measures in the study. Ethical approval was obtained from Royal Holloway, University of London’s Research Ethics Committee.

The final sample consisted of 24 adolescents aged between 12 and 18 (M = 13.8, *SD* = 2.30), including 21 males and 3 females. Mean verbal IQ, as measured by the Wechsler Abbreviated Scale of Intelligence (WASI; Wechsler 2008) verbal subset was 109.50 (*SD* = 19.01, range 70–147). All participants had a formal diagnosis of an ASC (including Asperger’s Syndrome) confirmed via parent report. The Social Communication Questionnaire (SCQ; Rutter et al. 2003), completed by parents, was used to confirm diagnoses; all participants scored at or above the cut-off score of 15 (M = 20.83, *SD* = 4.12, range = 15–27). Age, gender and verbal IQ were controlled for in the analyses due to their potential influence on identity development (Carrington & Graham 2001; Lai et al. 2015; Grotevant 1987; Braverman 2016).

### Materials and Procedure

Each participant took part in a 40–60 min session which began with the researcher explaining that some of the questions would address sensitive subject areas, and that participants should not feel obliged to answer any questions that made them feel uncomfortable. The researcher emphasised that all responses would be kept anonymous and confidential. Visual cards illustrating the words ‘Stop’, ‘Pause’ and ‘Take a Break’ were presented to participants on a desk in front of them, which they could use at any time. After the WASI verbal subsets, the following measures were administered in the order presented below.

#### Twenty Statements Task (TST)

The TST (Kuhn & McPartland 1954) is a measure used to assess how individuals define themselves using their own words. The task requires participants to respond to the question ‘Who am I?’ by writing down up to 20 statements beginning with “I am”, in a way that best defines their identity (e.g. ‘I am a thrill-seeker’, ‘I am autistic’). Each response was coded according to a coding scheme used by Rhee et al. (1995), which provided three separate scores for each participant: identity strength, identity complexity and identity quality. Identity strength was the total number of statements produced, up to a maximum of 20. Identity complexity was the number of different categories produced, out of a possible seven (social identity, physical descriptions, personal identity, personal qualities, interests and activities, relationships and environment). Identity quality was the proportion of specific items (i.e. qualified with some specific detail; e.g. ‘I am a brother’) divided by the total number of items reported. Marcia’s (1980) identity theory posits that well-developed identity comprises of a sense of one’s strengths and weaknesses (captured by identity strength and complexity scores), and one’s individual uniqueness (captured by identity quality score), therefore all three scores were included in the analysis to capture personal identity.

Nonsense responses (e.g. “playing around in circles”) and repeated responses were not coded. Following consultation with an autistic adult about how best to capture the responses of the TST, the percentage of positive and negative statements produced in each TST were also calculated. An additional coding scheme was therefore developed by the researchers to identify positive (e.g. ‘I am good at…’), negative (e.g. ‘I am not good at…’, ‘I am a failure’) and neutral statements (e.g. statements which have neither positive or negative connotations, or the connotation is unclear, such as ‘I am autistic’, ‘I am a dancer’). All statements were coded by two independent raters who met and agreed all coding. Inter-rater correlations were calculated for all of the component TST scores, with the resulting coefficients ranging from 0.71 to .93, with scores for identity strength, complexity and quality similar to those reported by Rhee et al. (1995; 0.76 to 1.00).

The TST has been used with adolescent cultural minority groups (Cousins 1989) and autistic adults (Tanweer et al. 2010). To the best of our knowledge, it has not yet been used with autistic adolescents, however, Carpenter and Meade-Pruitt (2008) note that its ease of use and simplicity mean it can be used across cultures and with varying participant groups.

#### Autism Identity Scale (AIS)

The AIS (Jarrett 2014) was developed based on the Deaf Acculturation Scale (DAS; Maxwell-McCaw & Zea, 2011). The AIS looks at whether an individual aligns more to an autistic or non-autistic culture, by asking questions related to cultural preference, knowledge, involvement and identification. The AIS consists of 32 items, with two 16-item scales (autistic (AIS1) and non-autistic (AIS2) acculturation). Each item was scored on a 5-point Likert scale (from ‘strongly disagree’ (1) to ‘strongly agree’ (5)). Example items from the AIS1 (autistic acculturation) scale include “Being autistic is an important part of who I am” and “I would prefer my closest friend(s) to have autism”. Example items from the AIS2 (non-autistic acculturation) scale include “I feel that I fit in with other people who do not have autism” and “I would prefer my education to be at a school with and without people with autism”. Jarrett (2014) tested the AIS with autistic adolescents on two occasions and found the measure to have moderate consistency over time (*r* = 0.50, p = .007); minor inconsistencies could be explained by the instability of the identity concept over time during adolescence (Klimstra et al. 2009). In the current study, internal consistency for AIS1 was acceptable (α = 0 .63) and good for AIS2 (α = 0.82). Half of the participants received the AIS1 first, and the other half received AIS2 first, which was randomly assigned to minimise order effects.

Following Jarrett (2014), data obtained from the AIS were organised into seven deciles, which were used to obtain cut-off scores to categorise each participant into one of four acculturation groups: marginalised (AIS1 score < 47, AIS2 score < 55), bicultural (AIS1 score > 48, AIS2 score > 56), assimilated (AIS1 score < 47, AIS2 score > 56) or separated (AIS1 score > 48, AIS2 score < 55). Group characteristics for the current sample are detailed in Table 1. No significant differences were found between groups on verbal IQ (F (3,23) = .12, p = .95) or age (F (3,23) = 1.31, p = .30).

**Table 1:** Sample characteristics for each acculturation group

	*n*	Age in years (mean, *SD*)	Verbal IQ (mean, *SD*)	Gender (male:female)
Marginalised (alignment to non-autistic culture)	7	13.00 (2.00)	105.71 (24.10)	6:1
Bicultural (alignment to both cultures)	7	14.57 (2.37)	111.57 (20.86)	6:1
Assimilated (alignment to neither culture)	7	14.29 (2.69)	110.29 (18.29)	6:1
Separated (alignment to autistic culture)	3	12.00 (.00)	111.67 (3.06)	3:0

#### Strengths and Difficulties Questionnaire (SDQ)

The SDQ (Goodman 1997) is a 25-item emotional and behavioural screening questionnaire. The self-report version, suitable for 11–18-year-olds, was administered to participants. Items are divided between five scales of five items each, generating scores for Behaviour Problems, Hyperactivity, Emotional Problems, Peer Problems and Prosocial Behaviours. The first four subscales are added to provide a Total Difficulties Score (internal reliability = .82; Goodman 2001), which was used in the current analyses as a measure of mental health as it was highly correlated with the emotional problems score (*r* (22) = .87, p < .001) and incorporates other aspects that contribute to mental health, such as behavioural problems and hyperactivity (Goodman 2001).

The SDQ has been shown to possess acceptable reliability and validity when assessing adaptation and mental health difficulties in children and adolescents (Goodman, 2001; Goodman & Goodman 2009). The SDQ has been used as a measure of mental health in typical adolescents (e.g. Ussher et al. 2007) and of psychiatric problems in autistic adolescents (Simonoff et al. 2013).

### Design and Data Analysis

An exploratory research design, which is predominantly utilised for discovery (Davies 2006) was used. The study was a cross-sectional, single group, correlational study. Hierarchical multiple regression was used to examine the relationship between personal identity (as measured by the TST strength, quality and complexity scores) and mental health (as measured by the SDQ). ANCOVA was used to explore the differences in mental health outcomes between acculturation types (as grouped by the AIS). Finally, MANCOVA was used to examine differences in positive/negative self-concepts (as measured by percentage positive and negative statements derived in the TST) between acculturation types. Verbal ability, as measured by the verbal tests of the WASI, age and gender were controlled for in all analyses. Data screening confirmed conditions for parametric testing were met (homogeneity of variance, normal distribution, and no extreme scores).

## Results

### Descriptive Statistics

The means and standard deviations of the SDQ scale scores, TST scores, including percentage positive and negative statements and the AIS scale scores (AIS1 = autistic scale, AIS2 = non-autistic scale) are presented in Table 2. The table includes the general population means for SDQ scale scores (Meltzer et al. 2000) to illustrate that the current sample had elevated levels of self-reported difficulties; there was a significant difference between the sample SDQ total difficulties score and the general population mean (*t* (23) = 5.92, p< .001). In line with previous research (e.g. Strang et al. 2012), 41.6% of the current sample of autistic adolescents had borderline or clinical levels of emotional and behavioural problems, as measured by the self-reported SDQ total difficulties score (scores greater than 16).

**Table 2:** Means, standard deviations and ranges for Strengths and Difficulties Questionnaire, the twenty statements task and the Autism Identity Scale

	Sample mean (SD)	General population mean
SDQ scale items		
Total difficulties	14.4 (6.01)	7.1
Emotional problems	3.8 (2.26)	1.6
Behavioural problems	2.3 (2.26)	1.3
Hyperactivity	4.9 (2.29)	2.8
Peer problems	3.4 (1.79)	1.4
Prosocial*	6.6 (2.28)	8.6
TST scores		
Identity strength	11.4 (4.77)	
Identity complexity	3.8 (1.55)	
Identity quality	0.8 (0.22)	
% Positive statements	36.8 (25.8)	
% Negative statements	19.3 (25.5)	
% Neutral statements	43.8 (23.5)	
AIS scale scores		
AIS1	46.8 (7.05)	
AIS2	56.2 (9.64)	

Range of SDQ scale scores: total difficulties = 0–40; emotional problems, behavioural problems, hyperactivity, peer problems, prosocial = 0–10. Range of TST scale scores: identity strength = number of ‘I am’ statements produced (0–20); identity complexity = number of categories (1–7); identity quality = specific items divided by total items (0–1). Range of AIS scores = 32–80

*Higher prosocial scores indicate better prosocial functioning

With the TST, although participants were asked to produce 20 statements, the average amount produced was 11.4 (*SD* = 4.77, range 4–20). Participants typically provided multiple categories in their identity descriptions, which also generally contained specific detail, rather than abstract statements with the mean identity complexity score (out of 7) being 3.79 (*SD* = 1.56, range 2–7). Identity quality (the proportion of specific items divided by the total number of items) was good overall (*M* = .80, *SD* = .22, range .25 – 1.00). More positive (36.8%, range 0–86%) than negative (19.3%, range 0–75%) statements were produced in the TST task; the remaining statements were neutral (43.8%, range 0–80%). Ten participants (42%) specifically mentioned autism in their TST responses. Average scores on the AIS2 were higher than the AIS1, indicating autistic adolescents typically felt more aligned to non-autistic, than autistic, culture (Table 2).

### Relationship Between Strength of Personal Identity and Mental Health

A hierarchical multiple regression was conducted using mental health (SDQ total difficulties score) as the outcome variable and age, gender and verbal IQ were controlled in the first step of the analysis, followed by personal identity (TST strength, complexity and quality) in the second step as predictors. Results of this analysis are reported in Table 3. The first step explained 6.8% of the variance in SDQ total difficulties score and was not a significant fit to the data (F (2, 23) = .48, p = .70). The second step explained 9.4% of the variance and was also not a significant fit to the data (F (6, 23) = .30, p= .93). None of the predictors significantly predicted SDQ total difficulties score (all *p*s > .53).

**Table 3:** Summary of hierarchical regression analysis for personal identity variables predicting mental health in autistic adolescents (*n* = 24)

	Step one			*p*	Step two			*p*
	B	SE B	β		B	SE B	β	
Age	− 26	.59	− .98	.67	− .27	.71	− .10	.71
Gender	3.59	4.16	.20	.40	3.35	5.26	.19	.53
Verbal IQ	− .02	.08	− .06	.81	− .02	.09	− .06	.82
TST strength					− .23	.39	− .18	.55
TST complexity					.67	1.44	.17	.64
TST quality					.52	10.45	.02	.96

### Relationship Between Acculturation Type and Mental Health

**Fig. 1:**
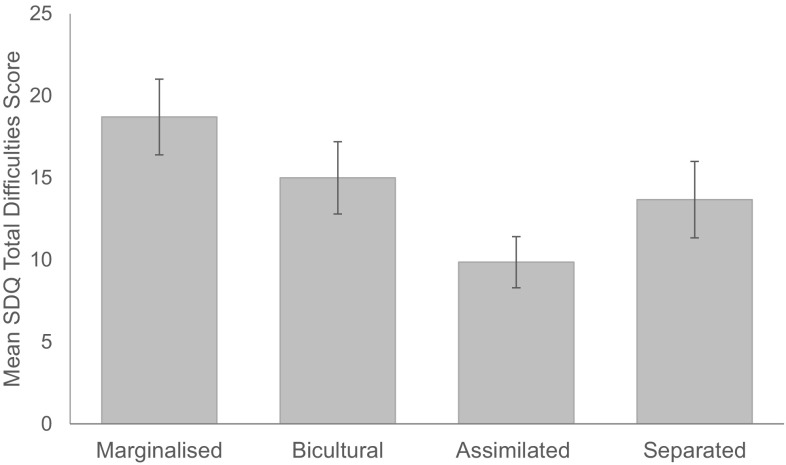
Mean SDQ total difficulties score according to acculturation group (marginalised = alignment to non-autistic culture; bicultural = alignment to both cultures; assimilated = alignment to neither culture; separated = alignment to autistic culture)

The means and standard errors for the four acculturation groups are displayed in Fig. 1. Those who aligned themselves more to neither non-autistic nor autistic culture (marginalised; *n* = 7) had the highest SDQ total difficulties scores, indicating poorer mental health, followed by those who aligned themselves to both autistic and non-autistic culture (bicultural; *n* = 7), those who aligned themselves only to autistic culture (separated; *n* = 3) and the lowest scores were found in those who aligned themselves only non-to autistic culture (assimilated; *n* = 7). A one-way ANCOVA was used to compare mean SDQ total difficulties scores in each acculturation group (marginalised, bicultural, assimilated and separated), controlling for age, gender and verbal IQ. The four groups did not differ significantly on SDQ total difficulties scores (F (3, 24) = 2.89, p= .066, η_p_^2^ = .34). There were no significant effects of the covariates (all *p*s > .31).

### Relationship Between Acculturation Type And Positive and Negative Self-Concept

**Fig. 2:**
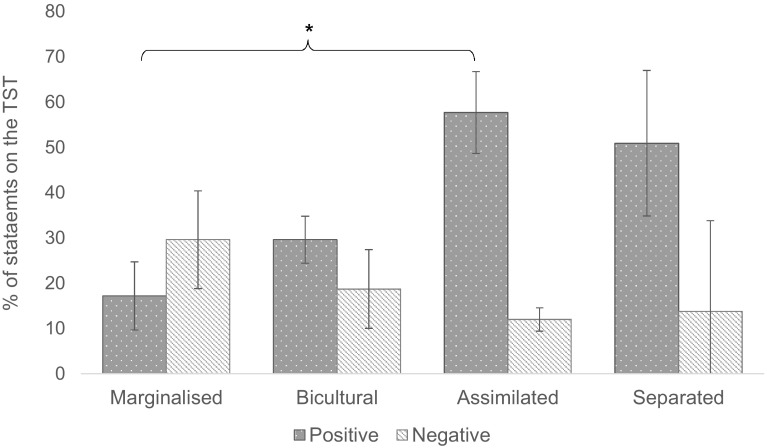
Mean percentage of positive and negative TST statements produced according to acculturation group (marginalised = alignment to non-autistic culture; bicultural = alignment no both cultures; assimilated = alignment to neither culture; separated = alignment to autistic culture). *p= .005

Figure 2 illustrates the mean percentage of positive and negative statements generated in each acculturation group. A one-way independent MANCOVA was used to compare the mean percentage scores of positive and negative statements generated from the TST about the self for each acculturation group, controlling for age, gender and verbal IQ. Using Pillai’s Trace, there no significant difference between acculturation groups on the percentage of positive and negative statements (*V* = .57, F (2,24) = 2.25, p= .062, η_p_^2^ = .28). Using Pillai’s Trace, there was a significant effect of the covariate verbal IQ on percentage of positive and negative scores between groups (*V* = .35, F (2, 24) = 4.23, p= .03). No other effects were significant (all *p*s > .14).

To further explore the trend between acculturation and the percentage of positive and negative statements, univariate ANOVAs on the percentage of positive and negative statements were conducted. These showed that there was a significant main effect of acculturation group only for the percentage of positive statements (F (3, 24) = 6.69, p= .003) but there was no significant main effect for percentage of negative statements (p = .54). Post hoc tests using Bonferroni corrections showed that the assimilated group generated a significantly higher proportion of positive statements about themselves than the marginalised group (57.7% vs. 17.2%; p= .005). No other results were significant. For the covariate verbal IQ, univariate ANOVAs found a significant main effect only for the percentage of positive statements (F (1, 24) = 6.40, p= .022).

## Discussion

The aim of the current study was to explore relationships between identity and mental health in autistic adolescents. No evidence was found to support the hypothesis that there would be a relationship between personal identity and mental health in autistic adolescents, using the Twenty Statements Task. Second, it was hypothesised that there would be differences between acculturation groups (marginalised, bicultural, assimilated and separated) on mental health scores, however this hypothesis was also not met. Finally, we predicted a relationship between acculturation type and positive or negative view of the self. There was a difference between groups on the proportion of positive, but not negative, statements produced about the self, with the marginalised group tending to generate fewer positive statements about themselves than the assimilated group. Specifically, aligning oneself only to non-autistic culture related to more positive statements about the self, than aligning to neither culture. These results should be treated with caution but suggest that aligning oneself more to one cultural group and committing to it, rather than aligning to neither group, may have benefits for positive self-concepts.

One of the aims of the current study was to contribute to understanding of the development of mental health difficulties amongst autistic adolescents. Developing a strong sense of personal identity (as measured by the TST), irrespective of autism, was not found to relate to mental health. Previous research has suggested that individuals might find it difficult to establish a strong self-concept if they have an awareness of being different (Corrigan et al. 2005; Link et al. 2014), which is something many autistic adolescents have reported (Humphrey & Lewis 2008). Thus, it is interesting to find that identity development did not have a negative impact on mental health in this group. Conclusions should be drawn carefully, however, due to the general subjectivity and complexity of measuring identity in research (Abdelal et al. 2009). The TST is a qualitative measure that is coded quantitatively, which has the potential to lessen the accuracy of analysis and miss some of the meaning and value gained in qualitative responses (Neuman 2014), although it has advantage in allowing for quantitative comparison with other variables. Coding schemes and multiple raters were used in an attempt to overcome issues of subjectivity and interpretation bias, by aiming to represent a reality within a certain threshold of consistency, accuracy, and attention to subjectivity and reflexivity of the researcher as instrument (Mays & Pope 2000).

Findings of the current study suggested that those who aligned only to non-autistic culture (assimilated) tended to generate more positive statements about themselves than those who aligned to neither culture. Marcia’s (1980) identity status theory could be used to explain such findings, whereby commitment refers to the degree of personal investment the individual expresses towards a certain identity (Kroger & Marcia 2011). Those in the separated or assimilated groups may have explored and committed to an identity, whereas those in the bicultural or marginalised groups (alignment to both or neither culture) may not yet have achieved strong commitment and thus an established sense of identity. Minority group research (e.g. Phinney 2003) supports this proposition, showing that exploring and committing to an ethnic identity can serve a protective role for individuals’ self-esteem; individuals who have explored and resolved issues pertaining to the group they identify with may feel more confident and have the tools to discuss issues regarding their group identity.

Alignment to autistic culture has been encouraged amongst the autistic community in recent years, via the neurodiversity movement, which advocates self-identification as autistic and the championing of neurological differences (Chamak et al. 2008; Ortega 2009). It is important to note a small number in the current sample aligned only to autistic culture, which might suggest that exploring and committing to autistic identity is rare amongst autistic adolescents, and may take time. This could be related to the relatively low mean age of the current sample (*M* = 13.8), especially given identity development is known to continue into young adulthood (Kroger et al. 2010a, b). Indeed, Cooper et al. (2017) found positive associations between autism identification and mental health in a sample of autistic adults.

Further, less than half of participants mentioned autism in their identity descriptions in the TST. There may be several reasons for this, and why more of these young people aligned themselves to non-autistic, rather than autistic, culture. Possible reasons include variation in exposure to other autistic individuals (Petalas et al. 2009), discourses in the home around autism (Dale et al. 2006), age of diagnosis (Coo et al. 2012) and personal perception of autism (Humphrey & Lewis 2008). Future longitudinal research should aim to examine this further, as it will help to build an understanding of how autistic identity development and acculturation manifest over time.

The finding that those who aligned to non-autistic culture had a more positive self-concept than those who aligned to neither culture shows support for previous group identity literature, which suggests group membership can have positive psychological consequences, for example by providing a sense of meaning, purpose and belonging (Haslam et al. 2009). Specifically, ethnic identity literature has highlighted that having a strong sense of social identity in adolescence can contribute to better self-esteem and fewer depressive symptoms (Umaña-Taylor and Updegraff 2007), which might help to explain why those who felt aligned to non-autistic culture had more positive self-concepts. Research on biculturalism has provided mixed findings, with some arguing that self-esteem and good psychological health are directly associated with minority group members’ sense of biculturalism (i.e. belonging to both the majority culture of their peers and their minority culture; Cornell and Lyness 2005). Others have found that those who feel caught between two cultures, feeling alienated from both cultures or feeling somewhat aligned to both, can develop mental health problems (e.g. Yeh 2003), which links to the current finding that those who aligned to neither culture (marginalised) had the least positive self-concepts. It should be noted, however, that most statements made in the TST in the current study had neutral connotations (44%), with 19% being negative and 37% positive. This finding likely reflects the way in which the self-concepts can be a complex myriad of different aspects of the self, not all of which have a negative or positive skew (Durkin, 1995).

Previous research examining the development of a sense of autistic social identity, or alignment to autistic culture, has been mixed, which is reflected in the current findings. For example, Carrington and Graham’s (2001) qualitative study indicated that not accepting autism as part of one’s identity could negatively affect an individual’s mental health, with some participants expressing a desire to ‘fit in’ but now knowing how. The current findings cannot contribute to understanding about whether some autistic adolescents take pride in being autistic (e.g. Cage et al. 2016) or whether autism identification can have positive effects on mental health and self-esteem (Cooper et al. 2017), given the low number of those who aligned only to autistic culture. However, our findings suggest that there is individual variation, with some adolescents aligning themselves to non-autistic culture. It is not known if members of this group hid or masked being autistic to fit in with the majority group, a concept known as “camouflaging” (Hull et al. 2017) which has been shown to be associated with poorer mental health (Bagatell 2007; Cage & Troxell-Whitman 2019). Assimilated participants may have accepted being autistic yet still preferred to align to non-autistic culture, potentially due to the pressures to ‘fit in’ with their neurotypical peers or to avoid bullying.

### Limitations

Caution must be taken when interpreting the findings due to the exploratory nature of the study and the small sample. The sample used is also at risk of bias as opt-in consent may have meant those with poorer mental health or identity issues were unlikely to agree to take part. Additionally, all participants attended mainstream secondary schools and therefore the experiences of those in alternative settings, such as specialist provisions or home education, are not explored, and these have been shown to vary in terms of outcomes of behaviour and socialisation for autistic adolescents (Reed et al. 2012).

Identity-based research has generated great interest, which has resulted in many ways of studying identity, including a variety of definitions of the concept and a wide variety of research methodologies. Two measures were used in the current study to investigate different aspects of identity, however both had methodological limitations and highlight the subjective nature of measuring such a broad construct. In particular, the AIS measure has not been widely used and the internal consistency for the autistic scale was only acceptable using the current sample, which raises issues of reliability. Furthermore, the method of analysis for this measure, suggested by Jarrett (2014), involved separating participants into four acculturation groups. This technique further reduced the sample sizes in each comparison group, which affects the validity and robustness of findings.

Lastly, there are issues with applying the acculturation model to the autistic population, despite strong supporting arguments presented by Myers et al. (2011), who make links between autistic and deaf minority groups, and describe the successful outcomes that have arisen using the acculturation model with deaf individuals. The model is based on feelings of alignment to majority and minority groups (Berry & Sabatier 2011) and the difficulties autistic individuals often have with social interaction and communication can impact on their ability to form relationships in groups (Foggo & Webster 2017). Therefore, lack of alignment to one or both groups could reflect a social communication difficulty rather than the individual’s cultural preference and how they identify themselves. There is also ongoing discussion as to whether autism should be considered a minority group, and Jaarsma and Welin (2012) argue independent autistic culture might be limited to those who are highly verbal, which warrants further exploration in future research. There is further controversy about whether beliefs and behaviours associated with neurological difference can be classified as ‘culture’ (Straus 2013). The conceptualisation of autistic culture used in the current study may be configured differently in future research, but the current findings nonetheless remain relevant to understanding identity processes for autistic adolescents. Despite the limitations outlined, the current study serves to highlight avenues for intervention and raise the profile of considering identity development and acculturation amongst autistic adolescents.

### Implications for Practice and Research

There are currently several initiatives to help autistic adolescents with their personal and social development, often delivered in school, including social skills development (see White et al. 2007 for a review) and more holistic approaches such as ILAUGH (Winner & Crooke 2009), which incorporates aspects such as developing conceptual processing and abstract thinking. However, few of these approaches address identity development, or actively help autistic adolescents to explore their personal and social identity preferences, to enable them to establish what autistic culture means to them.

Myers et al. (2011) recommend that individuals should be encouraged to explore their autistic identity when appropriate, rather than having to hide their autism and present as neurotypical in aspects of their life. This has been supported by adult autism literature, showing that lack of acceptance from others significantly predicts higher rates of stress and depression in autistic adults as well as experiences of “camouflaging” (Cage et al. 2018). The current findings suggest there are likely to be individual differences between acculturation preference and how adaptive that alignment is, however it appears that alignment to one culture may be more adaptive than aligning to neither autistic or non-autistic culture. Autistic adolescents should therefore be helped to explore their identity, through exposure to both groups, and by providing knowledge and information, necessary to form a cultural preference. This could be done in school, at home and with support from specialist autism services.

Identity development is not a fixed process; it is likely to be fluid and developed over time (Weinreich & Saunderson 2005). Therefore, this field of research would benefit from longitudinal data, mapping identity development and acculturation over time, perhaps from a starting point of receiving an autism diagnosis. Documenting mental health symptoms alongside this, using a large sample, would help to bolster the current findings and further explore the relationship between these concepts. The current findings give rise to more questions, which is to be expected in exploratory research (Stebbins 2001), providing a good starting point for further research into this topic area. Replication of these finding in different settings, with more female participants and with a large overall sample, is essential.

## Conclusion

Autistic adolescents face the task of working out who they are and where they fit in, in relation to their autistic and non-autistic peers. There was no evidence to suggest personal identity development, in terms of identity strength, complexity and quality, related to mental health difficulties in this group. In relation to acculturation, assimilated autistic adolescents tended to have a more positive self-concept than marginalised autistic adolescents. Further research is needed to strengthen and build upon these exploratory findings. However, they have important implications for the development of strategies to help autistic adolescents explore autistic and non-autistic culture and to commit to a preferred group identity, which may benefit their mental health and positive self-concept.

## Footnotes

1 We use identity-first language in this paper to reflect the preferences of the autism community (Kenny et al. 2016).

## References

[CR1] Abdelal R, Herrera YM, Johnston AI, McDermott R (2009). *Measuring identity: A guide for social scientists*.

[CR2] Acker L, Knight M, Knott F (2018). ‘Are they just gonna reject me?’ Male adolescents with autism making sense of anxiety: An Interpretative Phenomenological Analysis. Research in Autism Spectrum Disorders.

[CR3] Adreon D, Stella J (2001). Transition to middle and high school: Increasing the success of students with Asperger syndrome. Intervention in School and Clinic.

[CR4] American Psychiatric Association (2013). *Diagnostic and Statistical Manual of mental disorders*.

[CR5] Bagatell N (2007). From cure to community: Transforming notions of autism. Ethos.

[CR6] Baines AD (2012). Positioning, strategizing, and charming: How students with autism construct identities in relation to disability. Disability & Society.

[CR7] Baumeister RF, Leary MR (1995). The need to belong: Desire for interpersonal attachments as a fundamental human motivation. Psychological Bulletin.

[CR8] Berry JW (1986). The acculturation process and refugee behavior. Refugee Mental Health in Resettlement Countries.

[CR151] Berry JW (2005). Acculturation: Living successfully in two cultures. International Journal of Intercultural Relations.

[CR10] Berry JW, Sabatier C (2011). Variations in the assessment of acculturation attitudes: Their relationships with mental health. International Journal of Intercultural Relations.

[CR11] Blaine B, Crocker J (1995). Religiousness, race, and psychological well-being: Exploring social psychological mediators. Personality and Social Psychology Bulletin.

[CR12] Braverman A (2016). *Verbal ability and identity development* (Unpublished doctoral dissertation).

[CR13] Cage E, Bird G, Pellicano L (2016). ‘I am who I am’: Reputation concerns in adolescents on the autism spectrum. Research in Autism Spectrum Disorders.

[CR14] Cage E, Di Monaco J, Newell V (2018). Experiences of autism acceptance and mental health in autistic adults. Journal of Autism and Developmental Disorders.

[CR15] Cage E, Troxell-Whitman Z (2019). Understanding the reasons, contexts and costs of camouflaging for autistic adults. Journal of Autism and Developmental Disorders.

[CR16] Cappadocia MC, Weiss JA, Pepler D (2012). Bullying experiences among children and youth with autism spectrum disorders. Journal of Autism and Developmental Disorders.

[CR17] Capps L, Sigman M, Yirmiya N (1995). Self-competence and emotional understanding in high-functioning children with autism. Development and Psychopathology.

[CR18] Carpenter S, Meade-Pruitt SM (2008). Does the twenty statements test elicit self-concept aspects that are most descriptive?. World Cultures eJournal.

[CR19] Carrasco MA, Barrington C, Kennedy C, Perez M, Donastorg Y, Kerrigan D (2017). ‘We talk, we do not have shame’: Addressing stigma by reconstructing identity through enhancing social cohesion among female sex workers living with HIV in the Dominican Republic. Culture, Health & Sexuality.

[CR20] Carrington S, Graham L (2001). Perceptions of school by two teenage boys with asperger syndrome and their mothers: A qualitative study. Autism: The International Journal of Research and Practice.

[CR21] Carter EW, Common EA, Sreckovic MA, Huber HB, Bottema-Beutel K, Gustafson JR, Hume K (2014). Promoting social competence and peer relationships for adolescents with autism spectrum disorders. Remedial and Special Education.

[CR22] Chamak B, Bonniau B, Jaunay E, Cohen D (2008). What can we learn about autism from autistic persons?. Psychotherapy and Psychosomatics.

[CR23] Coo H, Ouellette-Kuntz H, Lam M, Yu C (2012). Correlates of age at diagnosis of autism spectrum disorders in six Canadian regions. Chronic Diseases and Injuries in Canada.

[CR24] Cooper K, Smith LG, Russell A (2017). Social identity, self-esteem, and mental health in autism. European Journal of Social Psychology.

[CR25] Cornell SL, Lyness KP (2005). Therapeutic implications for adolescent deaf identity and self-concept. Journal of Feminist Family Therapy.

[CR26] Corrigan PW, Kerr A, Knudsen L (2005). The stigma of mental illness: Explanatory models and methods for change. Applied and Preventive Psychology.

[CR27] Côté JE (2018). The enduring usefulness of Erikson’s concept of the identity crisis in the 21st century: An analysis of student mental health concerns. Identity.

[CR28] Cousins SD (1989). Culture and selfhood in Japan and the U.S. Journal of Personality and Social Psychology.

[CR29] Crocetti E, Klimstra TA, Hale WW, Koot HM, Meeus W (2013). Impact of early adolescent externalizing problem behaviors on identity development in middle to late adolescence: A prospective 7-year longitudinal study. Journal of Youth and Adolescence.

[CR30] Crocetti E, Rubini M, Luyckx K, Meeus W (2008). Identity formation in early and middle adolescents from various ethnic groups: From three dimensions to five statuses. Journal of Youth and Adolescence.

[CR31] Dale E, Jahoda A, Knott F (2006). Mothers’ attributions following their child’s diagnosis of autistic spectrum disorder: Exploring links with maternal levels of stress, depression and expectations about their child’s future. Autism.

[CR32] Davidson J (2008). Autistic culture online: virtual communication and cultural expression on the spectrum. Social & Cultural Geography.

[CR33] Davidson J (2008). Autistic culture online: virtual communication and cultural expression on the spectrum. Social & Cultural Geography.

[CR34] Davies P (2006). *Exploratory research*.

[CR152] Dekker, M. (1999). *On our own terms: Emerging autistic culture*. Paper presented at the Conferencia En Línea.

[CR35] Durkin K (1995). Developmental social psychology From infancy to old age.

[CR36] Erikson E (1968). *Youth: Identity and crisis*.

[CR37] Fiske ST (2009). *Social beings: Core motives in social psychology*.

[CR38] Foggo RSV, Webster AA (2017). Understanding the social experiences of adolescent females on the autism spectrum. Research in Autism Spectrum Disorders.

[CR39] Ghaziuddin M, Ghaziuddin N, Greden J (2002). Depression in persons with autism: Implications for research and clinical care. Journal of Autism and Developmental Disorders.

[CR40] Gill CJ (1997). Four types of integration in disability identity development. Journal of Vocational Rehabilitation.

[CR41] Gobbo K, Shmulsky S (2016). Autistic identity development and postsecondary education. Disability Studies Quarterly.

[CR42] Goodman R (1997). The strengths and difficulties questionnaire: A research note. Journal of Child Psychology and Psychiatry.

[CR43] Goodman R (2001). Psychometric properties of the strengths and difficulties questionnaire. Journal of the American Academy of Child and Adolescent Psychiatry.

[CR44] Goodman A, Goodman R (2009). Strengths and difficulties questionnaire as a dimensional measure of child mental health. Journal of the American Academy of Child and Adolescent Psychiatry.

[CR45] Grotevant HD (1987). Toward a process model of identity formation. Journal of Adolescent Research.

[CR46] Halpern, C. A. (1996). Listening in on Deaf culture. *University of Colorado Boulder*. Reprinted by permission from *Diversity and Distinction,* Harvard University. Retrieved from http://www.colorado.edu/journals/standards/V5N2/AWARD/halpern2.html

[CR47] Haslam SA, Jetten J, Postmes T, Haslam C (2009). Social identity, health and well-being: An emerging agenda for applied psychology. Applied Psychology.

[CR48] Holland D (2001). *Identity and agency in cultural worlds*.

[CR49] Hull L, Petrides K, Allison C, Smith P, Baron-Cohen S, Lai M, Mandy W (2017). “Putting on my best normal”: Social camouflaging in adults with autism spectrum conditions. Journal of Autism and Developmental Disorders.

[CR50] Humphrey N, Lewis S (2008). ‘Make me normal’: The views and experiences of pupils on the autistic spectrum in mainstream secondary schools. Autism: The International Journal of Research and Practice.

[CR51] Huws J, Jones R (2010). ‘They just seem to live their lives in their own little world’: Lay perceptions of autism. Disability & Society.

[CR52] Islam G (2014). Social identity theory. *Encyclopedia of critical psychology*.

[CR53] Jaarsma P, Welin S (2012). Autism as a natural human variation: Reflections on the claims of the neurodiversity movement. Health Care Analysis.

[CR54] Jarrett, H. (2014). An exploration of identity formation in autistic adolescents, its relationship with mental health, and the role of mainstream education provision in the identity formation process. (Unpublished doctoral thesis). University of Exeter, Exeter.

[CR55] Kenny L, Hattersley C, Molins B, Buckley C, Povey C, Pellicano E (2016). Which terms should be used to describe autism? Perspectives from the UK autism community. Autism.

[CR56] Klimstra TA, Hale WW, Raaijmakers QA, Branje SJ, Meeus WH (2009). Maturation of personality in adolescence. Journal of Personality and Social Psychology.

[CR57] Kroger J, Marcia JE (2011). The identity statuses: Origins, meanings, and interpretations. *Handbook of identity theory and research*.

[CR58] Kroger J, Martinussen M, Marcia JE (2010). Identity status change during adolescence and young adulthood: A meta-analysis. Journal of Adolescence.

[CR59] Kroger J, Martinussen M, Marcia JE (2010). Identity status change during adolescence and young adulthood: A meta-analysis. Journal of Adolescence.

[CR60] Kuhn MH, McPartland TS (1954). An empirical investigation of self-attitudes. American Sociological Review.

[CR61] Lai M, Lombardo MV, Auyeung B, Chakrabarti B, Baron-Cohen S (2015). Sex/gender differences and autism: Setting the scene for future research. Journal of the American Academy of Child and Adolescent Psychiatry.

[CR62] Lecavalier L (2006). Behavioral and emotional problems in young people with pervasive developmental disorders: Relative prevalence, effects of subject characteristics, and empirical classification. Journal of Autism and Developmental Disorders.

[CR63] Link BG, Phelan JC, Hatzenbuehler ML (2014). Stigma and social inequality. *Handbook of the social psychology of inequality*.

[CR65] Marcia JE (1980). Identity in adolescence. Handbook of Adolescent Psychology.

[CR66] Maxwell-McCaw D, Zea MC (2011). The deaf acculturation scale (DAS): Development and validation of a 58-item measure. Journal of Deaf Studies and Deaf Education.

[CR67] Mays N, Pope C (2000). Qualitative research in health care. Assessing quality in qualitative research. BMJ (Clinical Research Ed.).

[CR68] McGovern CW, Sigman M (2005). Continuity and change from early childhood to adolescence in autism. Journal of Child Psychology and Psychiatry.

[CR69] Meeus W, Iedema J, Helsen M, Vollebergh W (1999). Patterns of adolescent identity development: Review of literature and longitudinal analysis. Developmental Review.

[CR70] Meeus W, van de Schoot R, Keijsers L, Branje S (2012). Identity statuses as developmental trajectories: A five-wave longitudinal study in early-to-middle and middle-to-late adolescents. Journal of Youth and Adolescence.

[CR71] Meltzer H, Gatward R, Goodman R, Ford T (2000). *The mental health of children and adolescents in Great Britain*.

[CR72] Myers JA, Ladner J, Koger SM (2011). More than a passing grade: Fostering positive psychological outcomes for mainstreamed students with autism. Journal of Developmental and Physical Disabilities.

[CR150] Neuman D (2014). Qualitative research in educational communications and technology: A brief introduction to principles and procedures. Journal of Computing in Higher Education.

[CR73] Ortega F (2009). The cerebral subject and the challenge of neurodiversity. Biosocieties.

[CR74] Ozonoff S, Dawson G, McPartland JC (2002). *A parent’s guide to Asperger syndrome and high*-*functioning autism: How to meet the challenges and help your child thrive*.

[CR75] Petalas MA, Hastings RP, Nash S, Dowey A, Reilly D (2009). “I like that he always shows who he is”: The perceptions and experiences of siblings with a brother with autism spectrum disorder. International Journal of Disability, Development and Education.

[CR76] Phinney JS, Chun K, Organista P, Martin G (2003). Ethnic identity and acculturation. *Acculturation: Advances in theory, measurement, and applied research*.

[CR77] Postmes T, Branscombe NR (2002). Influence of long-term racial environmental composition on subjective well-being in African Americans. Journal of Personality and Social Psychology.

[CR78] Ratner K, Berman SL (2015). The influence of autistic features on identity development in emerging adults. Emerging Adulthood.

[CR79] Reed P, Osborne LA, Waddington EM (2012). A comparative study of the impact of mainstream and special school placement on the behaviour of children with autism spectrum disorders. British Educational Research Journal.

[CR80] Rhee E, Uleman JS, Lee HK, Roman RJ (1995). Spontaneous self-descriptions and ethnic identities in individualistic and collectivistic cultures. Journal of Personality and Social Psychology.

[CR81] Ritchie RA, Meca A, Madrazo VL, Schwartz SJ, Hardy SA, Zamboanga BL (2013). Identity dimensions and related processes in emerging adulthood: Helpful or harmful?. Journal of Clinical Psychology.

[CR82] Rosenthal M, Wallace GL, Lawson R, Wills MC, Dixon E, Yerys BE, Kenworthy L (2013). Impairments in real-world executive function increase from childhood to adolescence in autism spectrum disorders. Neuropsychology.

[CR83] Rutter M, Bailey A, Lord C (2003). *SCQ. The Social Communication Questionnaire*.

[CR84] Sam DL, Berry JW (2010). Acculturation: When individuals and groups of different cultural backgrounds meet. Perspectives on Psychological Science.

[CR85] Simonoff E, Jones CR, Baird G, Pickles A, Happé F, Charman T (2013). The persistence and stability of psychiatric problems in adolescents with autism spectrum disorders. Journal of Child Psychology and Psychiatry.

[CR86] Stebbins RA (2001). *Exploratory research in the social sciences*.

[CR87] Sterzing PR, Shattuck PT, Narendorf SC, Wagner M, Cooper BP (2012). Bullying involvement and autism spectrum disorders: Prevalence and correlates of bullying involvement among adolescents with an autism spectrum disorder. Archives of Pediatrics and Adolescent Medicine.

[CR88] Strang JF, Kenworthy L, Daniolos P, Case L, Wills MC, Martin A, Wallace GL (2012). Depression and anxiety symptoms in children and adolescents with autism spectrum disorders without intellectual disability. Research in Autism Spectrum Disorders.

[CR89] Straus JN (2013). Autism as culture. *The disability studies reader*.

[CR90] Tajfel H, Turner JC (1979). An integrative theory of intergroup conflict. The Social Psychology of Intergroup Relations.

[CR91] Tantam D (2000). Psychological disorder in adolescents and adults with Asperger Syndrome. Autism.

[CR92] Tanweer T, Rathbone CJ, Souchay C (2010). Autobiographical memory, autonoetic consciousness, and identity in Asperger syndrome. Neuropsychologia.

[CR93] Umaña-Taylor AJ, Updegraff KA (2007). Latino adolescents’ mental health: Exploring the interrelations among discrimination, ethnic identity, cultural orientation, self-esteem, and depressive symptoms. Journal of Adolescence.

[CR94] Ussher MH, Owen CG, Cook DG, Whincup PH (2007). The relationship between physical activity, sedentary behaviour and mental health among adolescents. Social Psychiatry and Psychiatric Epidemiology.

[CR95] Vickerstaff S, Heriot S, Wong M, Lopes A, Dossetor D (2007). Intellectual ability, self-perceived social competence, and depressive symptomatology in children with high-functioning autistic spectrum disorders. Journal of Autism and Developmental Disorders.

[CR96] Wang CW, Neihart M (2015). Academic self-concept and academic self-efficacy: Self-beliefs enable academic achievement of twice-exceptional students. Roeper Review.

[CR97] Waterman AS, Schwartz SJ, Zamboanga BL, Ravert RD, Williams MK, Agocha VB (2010). The Questionnaire for Eudaimonic Well-being: Psychometric properties, demographic comparisons, and evidence of validity. The Journal of Positive Psychology.

[CR98] Wechsler D (2008). Wechsler Adult Intelligence Scale-Fourth Edition (WAIS–IV).

[CR99] Weinreich P, Saunderson W (2005). *Analysing identity: Cross*-*cultural, societal and clinical contexts*.

[CR100] White SW, Keonig K, Scahill L (2007). Social skills development in children with autism spectrum disorders: A review of the intervention research. Journal of Autism and Developmental Disorders.

[CR101] Winner MG, Crooke PJ (2009). Social thinking: A training paradigm for professionals and treatment approach for individuals with social learning/social pragmatic challenges. SIG 1 Perspectives on Language Learning and Education.

[CR102] Yeh CJ (2003). Age, acculturation, cultural adjustment, and mental health symptoms of Chinese, Korean, and Japanese immigrant youths. Cultural Diversity and Ethnic Minority Psychology.

